# Characterization of co-infections of haemosporidian parasites in Swinhoe**’**s pheasant (*Lophura swinhoii*): Utilizing nanopore sequencing for species-level detection and mitochondrial-genome analysis

**DOI:** 10.1016/j.crpvbd.2025.100313

**Published:** 2025-08-27

**Authors:** Peihang Hong, Sijia Yu, Hau-You Tzeng, Yu-Hsuan Lin, Chao-Min Wang, Chung-Hung Lai, Shyun Chou

**Affiliations:** a Department of Veterinary Medicine, College of Veterinary Medicine, National Chung Hsing University, 145 Xing Da Rd., South District, Taichung City, Taiwan; b Graduate Institute of Microbiology and Public Health, College of Veterinary Medicine, National Chung Hsing University, 145 Xing Da Rd., South District, Taichung City, Taiwan; c WildOne Wildlife Conservation Association, 126 Xinxing, Chishang Township, Taitung County, Taiwan; d Department of Veterinary Medicine, National Chiayi University, 580 Xin Min Rd., Chiayi City, Taiwan

**Keywords:** Avian haemosporidian parasites, Co-infection, *Lophura swinhoii*, Mitochondrial genome assembly, Nanopore sequencing, Phylogenetic reconstruction

## Abstract

Avian haemosporidian parasites are vector-borne apicomplexans that infect bird species globally and pose considerable challenges in detection due to frequent co-infections and morphological convergence. In the present study, we first used Oxford Nanopore Technologies (ONT) to resolve co-infections of haemosporidians in Swinhoe’s pheasant (*Lophura swinhoii*), an island-endemic galliform. Blood smears revealed two morphologically distinct gametocyte forms: roundish and circumnuclear, and molecular analyses identified three mitochondrial lineages: two novel *Haemoproteus* lineages (hLOPSWI01 and hLOPSWI02) and one *Plasmodium* lineage (pNILSUN01). Phylogenetic reconstruction of mitogenomes resolved hLOPSWI01 and hLOPSWI02 within the *Parahaemoproteus* clade, whereas pNILSUN01 clustered in the *Giovannolaia-Haemamoeba* clade. Overall, this study revealed the efficacy of ONT in resolving cryptic co-infections through unfragmented mitogenome assembly, overcoming ambiguities inherent to Sanger sequencing. Our findings establish baseline haemosporidian diversity in *L. swinhoii* and highlight the necessity of combining long-read genomics with morphological scrutiny for accurate parasite taxonomy, particularly in understudied avian hosts facing conservation threats.

## Highlights

• Nanopore sequencing of haemosporidian co-infections for species-level resolution.
• Two novel *Haemoproteus* lineages (hLOPSWI01 and hLOPSWI02) identified in *Lophura swinhoii.*
• *Plasmodium* lineage pNILSUN01 demonstrates cross-order host transmission.
• Integration of morphology with long-read genomics advances parasite taxonomy.

## Graphical abstract

**Figure:**
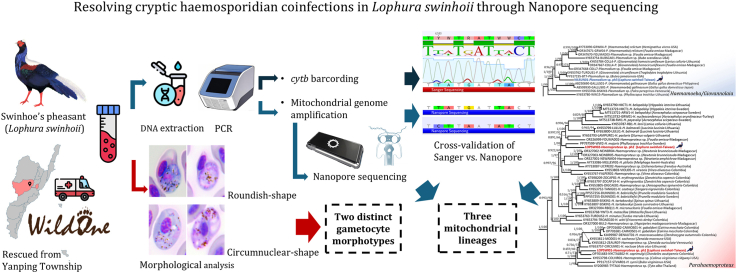

## 1 Introduction

Avian haemosporidian parasites (Haemosporida: Apicomplexa), comprising four genera, *Plasmodium*, *Fallisia*, *Haemoproteus*, and *Leucocytozoon* (Valkiunas, 2004; Pacheco and Escalante, 2023), are globally distributed dipteran-vectored hematozoa that infect > 50 % of the avian species across diverse ecosystems in non-polar regions (Clark et al., 2014; Rivero and Gandon, 2018; Santiago-Alarcon and Marzal, 2020). These parasites have catalyzed advances in parasitological medicine as well as the understanding of evolutionary ecology and host-pathogen dynamics as proxies for human malarial studies (Valkiunas, 2004; Santiago-Alarcon and Marzal, 2020). Therefore, the taxonomy and systematics of these parasites must be studied to enhance our understanding of the evolution and ecology of these species.

Studies have increasingly included molecular methods to investigate the prevalence and genetic diversity of avian haemosporidians (Bensch and Hellgren, 2020). The cytochrome *b* (*cytb*) barcode is pivotal for identifying haemosporidians and has been used to assign genetic lineage names that are recorded in the MalAvi database (Bensch et al., 2009). The database contains nearly 5000 lineages as of October 2024, providing insights into the DNA sequence variation and diversification of avian haemosporidians. This variation indicates the presence of mixed infections and/or co-infections in an avian host (Ricklefs and Fallon, 2002; Valkiūnas et al., 2003; Hellgren et al., 2004). Individual wild birds are often infected with two or more different parasites (Valkiunas, 2004; Valkiūnas et al., 2006). However, traditional methods such as *cytb* amplification and direct Sanger sequencing are unable to separate the infections in co-infected individuals, often resulting in jumbled chromatograms and double base-calling (Marzal et al., 2008; Martínez et al., 2009; Silva-Iturriza et al., 2012). In addition, conventional polymerase chain reaction (PCR) amplifies DNA sequences for species with higher DNA concentrations or DNA sequences that more closely match the primers, potentially masking the presence of co-infections (Bernotienė et al., 2016). Even multiplex PCR primers cannot detect mixed infections with haemosporidians of the same genus (Ciloglu et al., 2019). An alternative approach for identifying co-infections in unknown or complex situations involves performing the tedious step of cloning barcode fragments and then separate sequencing (Pérez-Tris and Bensch, 2005). Next-generation sequencing-based barcoding methods could be applied to identify co-infections (Yeo et al., 2022). However, these methods are limited by the small size of the *cytb* gene fragment, which reduces the number of informative phylogenetic sites and affects phylogenetic reconstructions and species delimitation (Ellis and Bensch, 2018; Pacheco et al., 2018a).

Phylogenetic analyses of the order Haemosporida have increasingly relied on mitochondrial genomes (Pacheco et al., 2020; Lotta-Arévalo et al., 2023; Valkiūnas et al., 2024) owing to the availability of a comprehensive dataset, which includes mitochondrial genomes from > 100 species of haemosporidians. These mitochondrial genomes help in increasing the resolution of phylogenetic analyses, which in turn resolves polytomies in the haemosporidian radiation in phylogenetic trees (Pacheco et al., 2018b). Conventional multifragment overlapping PCR is recommended for amplifying the mitochondrial genome in single-infection samples to ensure specificity and mitigate chimeric sequence artefacts (Musa, 2023). Long-range PCR and cloning are required for co-infected samples (Vieira et al., 2023). The sequences of the cloned PCR products contain polymerase errors, thereby requiring analysis of multiple clones to verify the correct sequence (Bensch and Hellgren, 2020). Although next-generation sequencing can amplify mitochondrial genomes (Karadjian et al., 2016; Ciloglu et al., 2020), the lack of reference genomes increases the risk of chimeras (Pacheco and Escalante, 2023). Therefore, long-read sequencing technologies have been developed to address the limitations of conventional PCR-dependent or short-read methods as robust approaches for handling the mitochondrial genome complexity in the separation of haemosporidian co-infections (Pacheco et al., 2024). The utility of these approaches is verified using PacBio HiFi sequencing by generating high-fidelity mitochondrial genomes across samples of mixed infections, thereby achieving single-read coverage that minimizes chimera formation and detects lineages even at low parasitemia (Pacheco et al., 2024). Oxford Nanopore Technologies (ONT) has emerged as a transformative platform for both resolving intricate genomic architectures of parasitic protozoans and enabling portable, real-time pathogen diagnostics. Its long-read sequencing capabilities can decipher complex structural genomic features across diverse parasitic taxa (Díaz-Viraqué et al., 2019; Liechti et al., 2019; Lee et al., 2021; Namasivayam et al., 2021; Menon et al., 2022; Higuera et al., 2023; Tetzlaff et al., 2024; Zhang et al., 2025). Beyond genome assembly, ONT shows clinical utility through its capacity to rapidly identify polyparasitic co-infections in animal reservoirs (Huggins et al., 2024a, 2024b; Jiménez et al., 2024; Matoute et al., 2024), while simultaneously supporting cost-effective molecular surveillance of antimalarial resistance. This is exemplified by targeted amplicon sequencing of *Plasmodium falciparum* drug-resistance loci in endemic regions, where platform portability and real-time data analysis enable timely public health interventions (Runtuwene et al., 2018; Girgis et al., 2023; De Cesare et al., 2024; Holzschuh et al., 2024).

Swinhoe’s pheasant (*Lophura swinhoii*) (family Phasianidae; order Galliformes) is a near-threatened species on the IUCN Red List and indigenous to Taiwan (Bird Life International, 2019) that exhibits the genetic island adaptation characteristics as an endemic island bird species (Xu et al., 2024). This phenomenon, referred to as island syndrome, leads to the reduced parasite resistance of these island hosts (Baeckens and Van Damme, 2020), potentially facilitating haemosporidian co-infection cascades. This study aimed to use Nanopore sequencing technology to separate complex co-infections of avian haemosporidians to detect and analyze their mitochondrial genomes at the species level. In addition, we aimed to fill the knowledge gap regarding avian haemosporidian infections in Swinhoe’s pheasant.

## 2 Materials and methods

### 2.1 Sample collection and blood film examination

The blood sample was obtained from an adult male Swinhoe’s pheasant that was rescued from Yanping Township (22°56′5″N, 121°1′58″E), Taitung, Taiwan, in March 2024. The bird was promptly transferred to WildOne Wildlife Conservation Association (https://www.wildonetaiwan.org/) for subsequent examinations and treatment. Approximately 1 ml of blood was collected from the brachial wing vein and stored in a lithium heparin tube until hematological examination. The blood smears were stained with Wright-Giemsa stain (BaSO Biotech, New Taipei, Taiwan) and observed using a Zeiss Axioscope 5 light microscope (ZEISS Group, Oberkochen, Germany). Photomicrographs were captured using a Tekfar Digital Camera (TEKFAR SCIENCE, Taichung, Taiwan), and digital measurements were conducted using ImageJ software (Schneider et al., 2012). The remaining blood was stored in a refrigerator (4–8 °C) until DNA extraction and molecular analysis.

### 2.2 DNA extraction and PCR screening

The genomic DNA was extracted from 100 μl of whole blood using a QIAamp® DNA Mini Kit (Qiagen, Hilden, Germany) and a taco™ mini-Automatic Nucleic Acid Extraction System (GeneReach, Taichung, Taiwan) that use magnetic bead separation technology. The PCR screening of haemosporidian parasites was based on a partial *cytb* gene (Hellgren et al., 2004). Positive PCR products were subsequently subjected to bidirectional Sanger sequencing provided by Genomics BioSci & Tech (New Taipei, Taiwan). Raw chromatograms underwent systematic processing using Geneious Prime 2023.2.1 (Biomatters Limited, Auckland, New Zealand, available at https://www.geneious.com), wherein base-calling errors were corrected using manual trace inspection and low-quality termini (Q-score < 20) were algorithmically trimmed. Consensus sequences derived from forward-reverse read alignments were authenticated through comparative analysis against the GenBank NCBI reference database (https://www.ncbi.nlm.nih.gov/genbank/) using the Megablast algorithm in Geneious Prime.

### 2.3 Mitochondrial genome amplification and ONT sequencing

The almost complete mitochondrial genome was amplified using KAPA HiFi HotStart ReadyMix (Roche Molecular Systems, Pleasanton, USA) with the primer sets AE170 and AE171. These primers are effective in amplifying the mitochondrial genomes (approximately 6 kb) of various haemosporidian species from vertebrate hosts (Pacheco et al., 2024). The sample DNA was diluted 100-fold for PCR amplification to minimize the influence of host’s DNA on subsequent high-throughput sequencing. PCR reactions were conducted in 20 μl volumes with the following conditions: initial denaturation at 95 °C for 3 min; followed by 35 cycles of 98 °C for 30 s, 69 °C for 30 s, and 72 °C for 3 min; and a final extension of 6 min at 72 °C. The PCR products were evaluated for subsequent sequencing using agarose gel electrophoresis. The resulting products were purified using AMPure XP Beads (Beckman Coulter, Indianapolis, USA) for further ONT sequencing. The total DNA concentration was measured using a Qubit 4.0 fluorometer (Thermo Fisher Scientific, Waltham, USA) and diluted to approximately 350 ng for library construction. First, end repair and dA-tailing were performed using a KAPA Hyper Prep kit (Roche Molecular Systems, Pleasanton, USA), and subsequent steps were conducted following the manufacturer’s protocol for a Native Barcoding Kit 24 V14 (SQK-NBD114.24, Oxford Nanopore Technologies, Oxford, UK). The sequencing library was loaded onto MinION Spot-on flow cell (FLO-MIN114 version R10.4.1). Real-time super-accuracy base-calling was conducted, and the trimming sequencing barcodes was automatic after base-calling in MinKNOW v24.06.8.

### 2.4 *De novo* assembly and sequence analysis

The raw data were primarily analyzed using Geneious Prime 2023.2.1. All reads underwent systematic processing commencing with quality filtration through BBDuk v38.84 (Q20 threshold) to eliminate low-confidence bases. The qualified reads were taxonomically contextualized through alignment against a curated mitochondrial reference database comprising 181 haemosporidian genomes (Pacheco et al., 2024) using Minimap2 v2.24 (Li, 2018) with a minimum divergence of 95 %, enabling genus-level classification based on conserved signature sequences. Genomic segments exceeding 2000 bp showing congruence with known haemosporidian genera were isolated, followed by removal of duplicates using Dedupe v38.84. *De novo* assembly was executed *via* Flye v2.9.1 (minimum overlap: 2000 bp) (Lin et al., 2016; Kolmogorov et al., 2019), with resultant contigs cross-validated against Sanger-derived sequences through MAFFT v7.490 (Katoh and Standley, 2013) implemented in Geneious Prime 2023.2.1 to distinguish potential multiple sequences.

### 2.5 Phylogenetic analysis

The mitochondrial genomes obtained were compared with the sequences available in the GenBank and MalAvi databases (Bensch et al., 2009) using BLAST implemented in Geneious Prime 2023.2.1. The genome was annotated using *P. falciparum* (M76611) as the reference genome (Feagin et al., 2012). The fragmented ribosomal RNAs and three protein-coding genes (cytochrome *c* oxidase 3 (*cox*3), cytochrome *c* oxidase 1 (*cox*1), and *cytb*) were identified.

A total of 110 avian haemosporidian mitochondrial genomes were retrieved from GenBank, encompassing three genera (*Plasmodium*, *Haemoproteus*, and *Leucocytozoon*). The non-protein coding region and the protein-coding genes exhibit a phylogenetic signal consistent with the previous mitogenomic phylogeny (Pacheco et al., 2018b, 2020; Lotta-Arévalo et al., 2023). Therefore, the alignment was performed using Clustal Omega v1.2.2 (Sievers et al., 2011) implemented in Geneious Prime 2023.2.1, which included these sequences and the three newly obtained mitochondrial sequences. The phylogenetic relationships were inferred from the alignment of partial mitochondrial genomes (5358 bp excluding gaps), which maintained their original order in the genome. Both Maximum Likelihood (ML) and Bayesian Inference (BI) methods were used to construct the phylogenetic tree. The substitution model was estimated for each method using ModelFinder v2.2.0 (Kalyaanamoorthy et al., 2017) implemented in PhyloSuite v1.2.3 (Zhang et al., 2020; Xiang et al., 2023). GTR+F+I+G4 was the best-fitting model for both methods based on the corrected Akaike information criterion (AIC) score. The BI phylogenies were inferred using MrBayes v3.2.7a (Ronquist et al., 2012) implemented in PhyloSuite v1.2.3. The Markov Chain Monte Carlo algorithm was run for 50 million generations, with sampling every 1000 generations, and the initial 25 % of the sampled data were discarded as “burn-in”. The ML phylogenies were executed with 1000 ultrafast (Minh et al., 2013) bootstraps using IQ-TREE v2.2.0 (Nguyen et al., 2015) implemented in PhyloSuite v1.2.3.

## 3 Results

### 3.1 Morphological characteristics

**Fig. 1:**
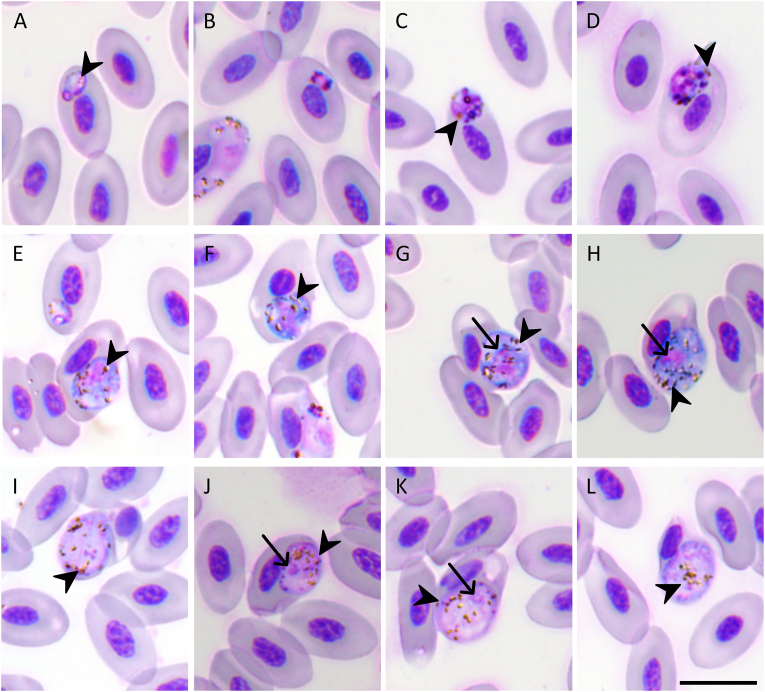
Haemosporidian parasites from the blood of Swinhoe’s pheasant (*Lophura swinhoii*). **A** Trophozoites. **B**-**D** Erythrocytic meronts. **E**-**H** Roundish macrogametocytes. **I**-**L** Roundish microgametocytes. Long arrows: parasite nuclei; arrowheads: pigment granules. Wright-Giemsa-stained thin blood films. *Scale bar*: 10 μm.

**Fig. 2:**
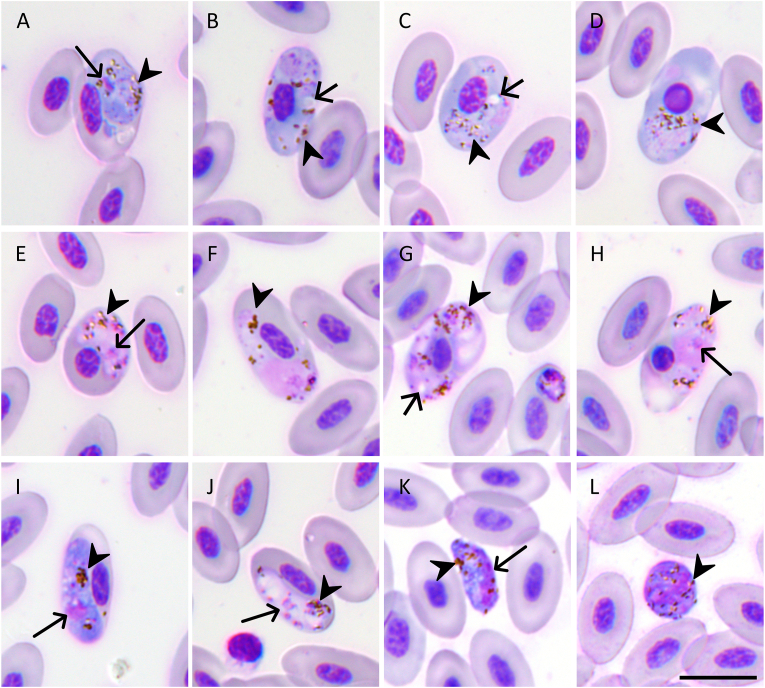
Haemosporidian parasites from the blood of Swinhoe’s pheasant (*Lophura swinhoii*). **A**-**D** Circumnuclear macrogametocytes. **E**-**H** Circumnuclear microgametocytes. **I**, **J** Microhalteridial gametocytes. **K**, **L** Putative macrogametes and/or zygotes. Long arrows: parasite nuclei; short arrows: vacuoles; arrowheads: pigment granules. Wright-Giemsa-stained thin blood films. *Scale bar*: 10 μm.

The gametocytes and meronts were identified based on standardized morphological criteria (Valkiūnas and Iezhova, 2018, 2022). Two distinct morphological forms of fully grown gametocytes were discernible: a roundish and a circumnuclear (Fig. 1, Fig. 2). The roundish, fully grown gametocytes induced substantial deformation of the infected erythrocytes, with pronounced displacement of the host cell nuclei (Fig. 1E–L). The cytoplasmic staining of these round-type gametocytes was darker than that of the circumnuclear forms. The circumnuclear gametocytes displayed a large vacuole (approximately 1.3 μm in diameter) (Fig. 2B and C) and morphologically altered the host erythrocytes, including the development of rounded nuclear conformations in some infected cells (Fig. 2D and H). In addition, microhalteridial-type gametocytes with an intensely stained vacuole-free cytoplasm were observed (Fig. 2I and J); however, their developmental status could not be determined due to the morphological variations that occur during gametocyte maturation. Putative macrogametes and/or zygotes were observed in the blood film (Fig. 2K and L), possibly because of the blood being collected and stored prior to being sent to the laboratory. *In vitro* development may have occurred during this period (Valkiūnas et al., 2021).

### 3.2 Molecular analyses and lineage identification

**Fig. 3:**
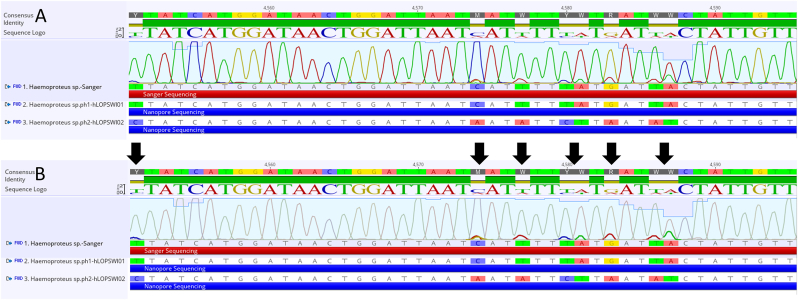
Alignment of sequences from Sanger sequencing and Nanopore sequencing. **A** Chormatogram of Sanger sequencing demonstrated complete concordance between Sanger sequencing reads and lineage hLOPSWI01. **B** Highlight of the nine positions of low-abundance double base-calling (*black arrows*), which are consistent with lineage hLOPSWI02.

A 539 bp sequence that aligned with *Haemoproteus* sp. lineage hOTULEM01 (98.12 % similarity) in the MalAvi database was produced using Sanger sequencing. The ambiguous chromatographic peaks (such as positions 4583G/A and 4588A/T; Fig. 3) reveal possible mixed infections, thereby necessitating ONT sequencing for resolving the haplotypes.

Sequencing on the MinION platform yielded 7343 reads (11 Mb total), of which 6744 and 54 were specifically mapped to *Haemoproteus* and *Plasmodium,* respectively, using Minimap2 v2.24. The long-read assemblies generated by Flye v2.9.1 showed genus-specific genomic signatures: three contigs were recovered, including two *Haemoproteus* spp. (pairwise identity 93.87 %; GenBank: LC867942, LC867943) and one *Plasmodium* sp. (GenBank: LC867944) contig. The results of BLASTn analysis against the MalAvi database (Bensch et al., 2009) identified the *Plasmodium* sequence as genetic lineage pNILSUN01 (GenBank: DQ659586). Both *Haemoproteus* lineages (hLOPSWI01 and hLOPSWI02) considerably diverged from the existing entries; LOP denoted host genus *Lophura* (Phasianidae) and SWI represented *swinhoii* based on the MalAvi guidelines, qualifying as novel genetic lineages. The results of cross-validation with the Sanger sequencing results revealed complete agreement between the conventional sequencing reads and hLOPSWI01, whereas the ONT reads resolved ambiguous base calls as a low-abundance hLOPSWI02 lineage (Fig. 3), thereby confirming a cryptic mixed infection.

### 3.3 Phylogenetic analysis of the avian haemosporidian mitochondrial genomes

**Fig. 4:**
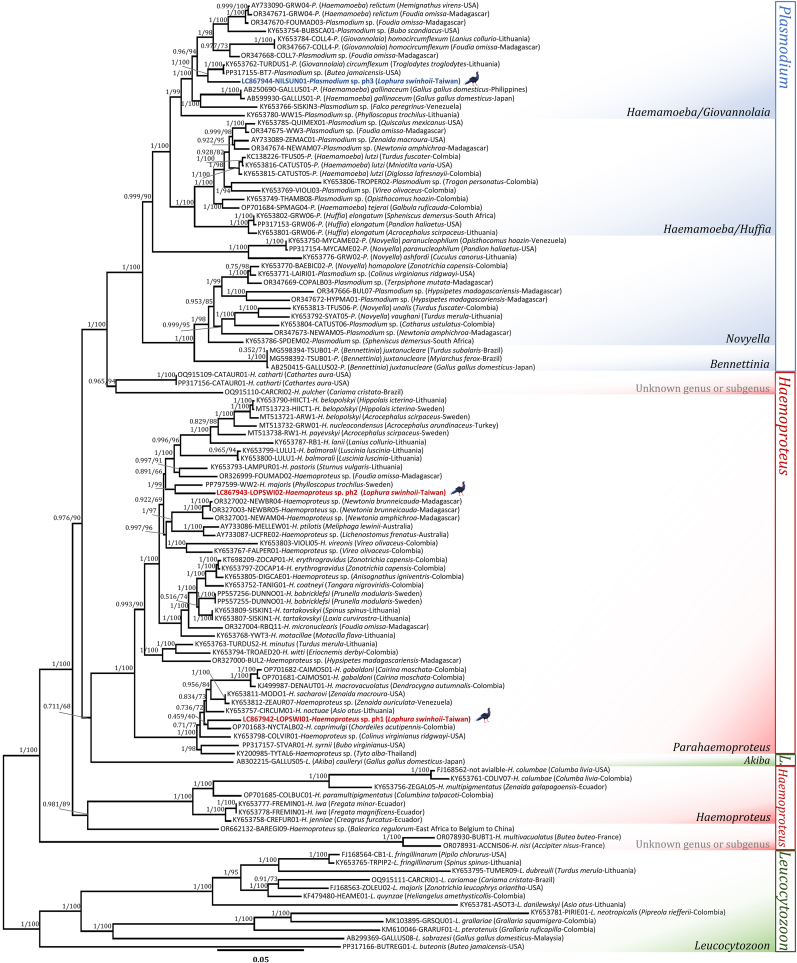
Phylogenetic reconstructions of avian haemosporidian using mitochondrial genomes. Bayesian Inference (BI) and Maximum Likelihood (ML) phylogenies were inferred using 110 partial mitochondrial genomes (5358 bp excluding gaps) belonging to three genera. The values near branches are BI posterior probabilities/ML bootstrap values, respectively. The new mitochondrial genomes obtained in this study are indicated in bold.

The phylogenetic reconstruction of the avian haemosporidian mitogenomes showed congruent topologies between the BI and ML analyses (Fig. 4). The genus *Leucocytozoon* exhibited polyphyly, with its subgenus *Leucocytozoon* forming a sister clade with other avian haemosporidians. The subgenus *Akiba* was basally located with subgenus *Parahaemoproteus* (BI posterior probability/ML bootstrap: 0.71/68). The genus *Haemoproteus* showed polyphyly; however, the monophyly of its two subgenera, *Haemoproteus* and *Parahaemoproteus*, was strongly supported, consistent with prior studies (Pacheco et al., 2018b; Lotta-Arévalo et al., 2023). The clade containing *H. multivacuolatus* and *H. nisi* (infecting accipitrid raptors in France) (Harl et al., 2024) basally diverged to the *Haemoproteus*-*Plasmodium* complex, with maximum statistical support (1/100). The genetic lineage hBAREGI09 (*Haemoproteus* sp. ex *Balearica regulorum*), a sister taxon to *H. antigonis* (Shen et al., 2024)*,* shared a common ancestor with the subgenus *Haemoproteus* (0.98/89). Both of the newly identified genetic lineages fell within the *Parahaemoproteus* clade: lineage hLOPSWI01 weakly aligned with the *H. caprimulgi* lineage hNYCTALB02 (ex *Chordeiles acutipennis*, Colombia; topology support: 0.46/40; patristic distance Δ = 0.032). Lineage hLOPSWI02 formed a strongly-supported clade with the *H. major* lineage hWW2 (ex *Phylloscopus trochilus*, Sweden; topology support: 1/99; patristic distance Δ = 0.038). Avian *Plasmodium* spp. resolved as a monophyletic group with distinct subclades, consistent with previously published taxonomic frameworks (Pacheco and Escalante, 2023). The identified *Plasmodium* sp. lineage pNILSUN01 clustered within the *Haemamoeba*-*Giovannolaia* clade, sharing ancestry with the *P.* (*Giovannolaia*) *circumflexum* lineage pTURDUS1 and *Plasmodium* sp. lineage pBT7 (topology support: 1/100; patristic distance Δ = 0.028).

## 4 Discussion

We successfully used ONT sequencing to resolve co-infections with avian haemosporidian parasites in Swinhoe’s pheasant, *L. swinhoii*, and identify three mitochondrial genomes. The integration of long-read sequencing overcame the limitations of traditional Sanger sequencing methods, which fail to separate co-infections in samples due to ambiguous chromatograms (Bernotienė et al., 2016). The capacity of ONT to generate mitochondrial contigs enabled species-level resolution, confirming cryptic co-infections of *Plasmodium* spp. lineage pNILSUN01 and two undescribed *Haemoproteus* lineages, named hLOPSWI01 and hLOPSWI02. This methodological advance aligns with efforts to leverage long-read sequencing for resolving complex haemosporidian assemblages (Pacheco et al., 2024).

The lineage pNILSUN01 (*Plasmodium* sp.), initially described in *Niltava sundara* (Passeriformes) from Myanmar (Beadell et al., 2006), has subsequently been detected across diverse passerine hosts in Asia (Ishtiaq et al., 2017; Menzies et al., 2021; Huang et al., 2022). These prior studies did not morphologically characterize this lineage. Our study documents the occurrence of the lineage pNILSUN01 in a galliform host (*L. swinhoii*), supporting the hypothesis that the host specificity of avian *Plasmodium* is low, showing cross-order transmission (Valkiūnas and Iezhova, 2018; Ciloglu et al., 2020). This characteristic sharply contrasts that of *Haemoproteus* spp., which typically reveal high host specificity and limited inter-order transmission (Valkiunas, 2004). Only six *Haemoproteus* spp. (*H. lophortyx*, *H. ammoperdix*, *H. rileyi*, *H. mansoni*, *H. pratasi*, and *H. stableri*) infecting galliform birds have been morphologically described in phasianid hosts (Valkiūnas and Iezhova, 2022). *Haemoproteus rileyi* was reported in the Taiwan bamboo partridge (*Bambusicola sonorivox*), another endemic Taiwanese species (Bennett and Peirce, 1989). However, the gametocyte morphology observed in our study diverges from previous descriptions of gametocyte morphology of *Haemoproteus* spp. described in phasianid hosts (Bennett and Peirce, 1989). In addition, to the best of our knowledge, no published study has used the associated genetic data to determine morphological and molecular taxonomy.

Molecular surveys of haemosporidian genetic lineages in hosts of the Phasianidae have been comprehensively investigated, with most studies concentrating on the domestic chicken (*Gallus gallus*). This host species has been documented to harbor one *Haemoproteus* lineage, 15 *Plasmodium* lineages, and 46 *Leucocytozoon* lineages (Bensch et al., 2009; Swangneat et al., 2025). However, sporadic molecular reports have been published for non-domestic phasianid hosts, predominantly involving *Plasmodium* spp. (Table 1). Haemosporidian studies on Phasianidae in Taiwan have exclusively relied on morphological methods (Manwell, 1962; Pan, 1963; Morii et al., 1986), leaving a gap regarding the molecular characterization of the parasites. We addressed this by obtaining molecular evidence of co-infections in the Swinhoe’s pheasant (*L. swinhoii*), thus highlighting the need to apply integrative taxonomic approaches in understudied avian hosts.

**Table 1:** Genetic lineages of haemosporidian parasites in non-domestic phasianid hosts.

Host species	Host subfamily	Parasite lineage	Location	Reference
*Crossoptilon crossoptilon*	Phasianinae	*Plasmodium juxtancleare* pGALLUS02	Japan (captive)	Murata et al. (2008)
*Pavo cristatus*	Phasianinae	*Plasmodium elongatum* pGRW06	Brazil (captive)	Chagas et al. (2017)
		*Plasmodium* sp. pDENVID01		
*Pavo muticus*		*Plasmodium* sp. pDENVID01		
*Lophura swinhoii*	Phasianinae	*Haemoproteus* sp. hLOPSWI01&02	Taiwan	This study
		*Plasmodium* sp. pNILSUN01		
*Peliperdix sephaena*	Perdicinae	*Plasmodium* sp. pPELSEP01-04	South Africa	Ndlovu et al. (2024)
Alaskan grouse and ptarmigan	Tetraoninae	*Haemoproteus* sp. hAKGPH01-05	USA	Smith et al. (2016)
		*Plasmodium* sp. pAKGPP01-03		
		*Leucocytozoon* sp. lAKGPL01-14		
Alaskan grouse and ptarmigan	Tetraoninae	*Haemoproteus* sp. hTETURO01-02	USA	De Amaral et al. (2023)
		*Plasmodium* sp. pBT7		
		*Leucocytozoon* sp. lCOLBF22, lCOLBF24, lAKGPL09, lAKGPL14, lLAGLAG02-04, lGALLUS25, lCYASTE01&02, lSPISEN05		
*Meleagris gallopavo*	Meleagridinae	*Leucocytozoon* sp. lGHA146	Ghana	Agbemelo-Tsomafo et al. (2023)
*Meleagris gallopavo*	Meleagridinae	*Plasmodium gallinaceum* pGALLUS01, pMELGAL	Thailand	Chatan et al. (2024)
		*Plasmodium juxtancleare* pGALLUS02		
		*Plasmodium* sp. pACCBAD01		

We identified two distinct gametocyte morphotypes in blood smears: roundish and circumnuclear types (Fig. 1, Fig. 2). Although three mitochondrial lineages were detected molecularly, the morphology of the third lineage (microhalteridial-type gametocytes) was not assigned (Fig. 2I and J). These microhalteridial forms may represent immature developmental stages of the observed roundish or circumnuclear gametocytes, as the morphology of immature gametocytes often differs from that of fully mature gametocytes (Valkiunas, 2004). Differentiating *Haemoproteus* and *Plasmodium* spp. based on gametocyte morphology is inherently challenging, as both genera exclusively develop within erythrocytes and produce haemozoin pigment granules (Valkiūnas and Iezhova, 2018, 2022).

Roundish gametocytes are rare among *Haemoproteus* spp., with fully mature forms definitively documented only in *H. ortalidum* (parasitizing Neotropical Galliformes) (Valkiunas, 2004) and *H. parus* (Passeriformes) (Bennett, 1989). The roundish gametocytes studied here differed morphologically from the macrogametocytes of *H. ortalidum*, which characteristically display a large, clear vacuole (Valkiunas, 2004). The genetic divergence (96 % identity) between the lineages we identified and *H. ortalidum* (GenBank: MW899346) further supports their distinct taxonomic status. The roundish gametocytes share morphological similarities with *H. parus*, originally described in *Parus bicolor* (Passeriformes) (Bennett, 1989), including deep cytoplasmic staining, scattered pigment granules, larger gametocyte dimensions, and erythrocyte deformation. However, *H. parus* had not been molecularly characterized. The subsequent morphological analyses indicated that this species belongs to *Plasmodium* (*Haemamoeba*) owing to overlapping features (Valkiunas, 2004; Valkiūnas and Iezhova, 2022). The roundish morphotype may correspond to *Plasmodium* lineage pNILSUN01, which clustered within the *Giovannolaia*-*Haemamoeba* clade in the phylogenetic tree in our study. These findings align with prior hypotheses positing misclassification of certain “roundish *Haemoproteus*” morphotypes as *Plasmodium* spp., particularly because of the association of the lineage with hosts of the Passeriformes (Valkiunas, 2004). Circumnuclear-type gametocytes positioning further complicates diagnostics, as this morphology occurs in *Plasmodium* (predominantly subgenus *Giovannolaia*) and *Haemoproteus* (subgenus *Parahaemoproteus*) (Valkiūnas and Iezhova, 2018, 2022). The molecular lineages phylogenetically corresponded to the *Giovannolaia-Haemamoeba* and *Parahaemoproteus* clades in our study, agreeing with the observed circumnuclear morphology. However, resolving the precise morpho-molecular linkages requires further sampling across multiple host individuals to account for potential developmental polymorphism and interspecific variation.

Long-read sequencing technologies can resolve complex haemosporidian co-infections, addressing the limitations of traditional Sanger sequencing and short-read approaches (Pacheco et al., 2024). These technologies generate unfragmented mitochondrial genomes, enabling precise haplotype phasing and accurate detection of co-infecting lineages, unlike short-read methods, which require fragment assembly and cannot resolve haplotypes in mixed infections (Pacheco and Escalante, 2023; Pacheco et al., 2024). Although PacBio HiFi sequencing has been validated for this purpose (Pacheco et al., 2024), the present study represents the first application of ONT in avian haemosporidian co-infection resolution. The real-time data streaming and adaptive sampling capabilities of ONT are advantageous in co-infection resolution compared with PacBio HiFi. The ability of ONT to dynamically adjust sequencing parameters during runs without prior library modification allows the targeted enrichment of low-abundance lineages, which is critical for comprehensive detection of the pathogens (Petersen et al., 2019; Shafin et al., 2020; Wang et al., 2021; De Meulenaere et al., 2024). In contrast, the circular consensus sequencing mode in PacBio lacks this adaptability, requiring fixed run durations and post-sequencing bioinformatic corrections (Wenger et al., 2019). Furthermore, the portability of ONT devices facilitates field-deployable sequencing in remote avian habitats, and the flexible run configurations and reusable flow cells of ONT substantially reduce the operational costs compared with those of PacBio (Wang et al., 2021; Girgis et al., 2023; Matoute et al., 2024; De Cesare et al., 2024; Holzschuh et al., 2024). However, we identified limitations in the primer affinity biases during mitochondrial genome amplification. The disproportionate read counts of *Haemoproteus* (6744 reads) *versus Plasmodium* (54 reads) indicate the preferential amplification of certain lineages. Such biases are similar to the challenges reported in conventional PCR assays, where lineage-specific primer affinities skew detection sensitivity (Bernotienė et al., 2016; Ciloglu et al., 2019). As such, future studies should prioritize optimizing multiplex PCR primer sets to achieve balanced amplification across genera or implementing direct nanopore adaptive sequencing to reduce host DNA interference and preferentially enrich parasite genomes. A combinatorial strategy using phased primer panels (De Cesare et al., 2024; Killander et al., 2025), validated through *in silico* binding affinity analysis and experimental co-infection models, may prevent or reduce amplification bias. Alternatively, nanopore adaptive sequencing protocols that selectively sequence haemosporidian DNA during real-time runs (De Meulenaere et al., 2024) could enhance mitochondrial genome recovery from mixed infections by avoiding PCR competition. Finally, a standardized multigene reference database across mitochondrial, apicoplast, and nuclear loci is required to reconcile morphological descriptions with molecular lineage diversity in understudied avian hosts.

## 5 Conclusions

This study revealed that ONT effectively resolves complex avian haemosporidian co-infections in Swinhoe’s pheasant (*L. swinhoii*), identifying two novel *Haemoproteus* lineages (hLOPSWI01 and hLOPSWI02) and a *Plasmodium* lineage (pNILSUN01) through mitochondrial genome assembly and phylogenetic reconstruction. By generating unfragmented mitochondrial genomes, ONT overcame the limitations of traditional Sanger sequencing that obscure mixed infections, whereas phylogenetic analyses validated cryptic lineage differentiation within morphologically ambiguous gametocytes. These findings emphasize the necessity of integrating long-read genomics with morphological validation to advance taxonomic accuracy in avian haemosporidians, particularly for conservation-priority species threatened by co-infection-driven pathogenicity. Overall, this study provides a methodological framework for resolving cryptic parasite diversity in island-endemic birds, informing both evolutionary ecology and wildlife disease management strategies.

## Ethical approval

Not applicable.

## CRediT authorship contribution statement

**Peihang Hong:** Conceptualization, Data curation, Methodology, Visualization, Writing – original draft, Writing – review & editing. **Sijia Yu:** Data curation, Methodology, Writing – original draft. **Hau-You Tzeng:** Conceptualization, Methodology, Writing – review & editing. **Yu-Hsuan Lin:** Methodology, Investigation. **Chao-Min Wang:** Conceptualization, Validation, Resources. **Chung-Hung Lai:** Resources, Supervision, Writing – review & editing. **Shyun Chou:** Conceptualization, Data curation, Methodology, Project administration, Writing – review & editing.

## Funding

This research did not receive any specific grant from funding agencies in the public, commercial, or not-for-profit sectors.

## Declaration of competing interests

The authors declare that they have no known competing financial interests or personal relationships that could have appeared to influence the work reported in this paper.

## Data Availability

The nucleotide sequences obtained in this study were deposited in the DNA Data Bank of Japan (DDBJ, http://www.ddbj.nig.ac.jp) with the following accession numbers: LC867942, LC867943 and LC867944.
